# An integrative approach for building personalized gene regulatory networks for precision medicine

**DOI:** 10.1186/s13073-018-0608-4

**Published:** 2018-12-19

**Authors:** Monique G. P. van der Wijst, Dylan H. de Vries, Harm Brugge, Harm-Jan Westra, Lude Franke

**Affiliations:** Department of Genetics, 5th floor ERIBA building, Antonius Deusinglaan 1, 9713AV Groningen, University of Groningen, University Medical Center Groningen, Groningen, The Netherlands

## Abstract

Only a small fraction of patients respond to the drug prescribed to treat their disease, which means that most are at risk of unnecessary exposure to side effects through ineffective drugs. This inter-individual variation in drug response is driven by differences in gene interactions caused by each patient’s genetic background, environmental exposures, and the proportions of specific cell types involved in disease. These gene interactions can now be captured by building gene regulatory networks, by taking advantage of RNA velocity (the time derivative of the gene expression state), the ability to study hundreds of thousands of cells simultaneously, and the falling price of single-cell sequencing. Here, we propose an integrative approach that leverages these recent advances in single-cell data with the sensitivity of bulk data to enable the reconstruction of personalized, cell-type- and context-specific gene regulatory networks. We expect this approach will allow the prioritization of key driver genes for specific diseases and will provide knowledge that opens new avenues towards improved personalized healthcare.

## Background

**Fig. 1:**
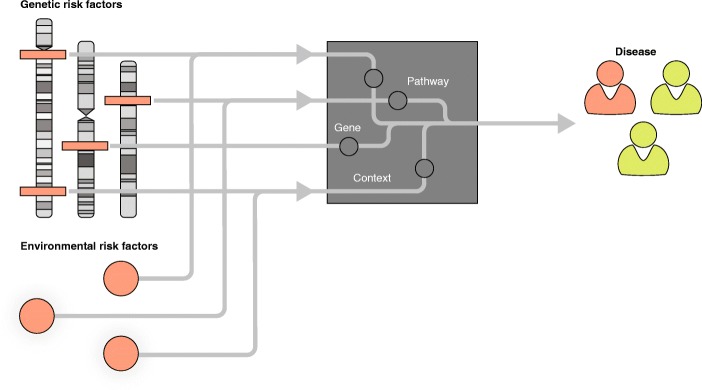
The link between genetic and environmental risk factors in disease. Understanding the interplay between genetic and environmental risk factors enables identification of the disease-associated context, causal genes, and pathways. This leads to a better understanding of why certain individuals become ill, whereas others do not

**Fig. 2:**
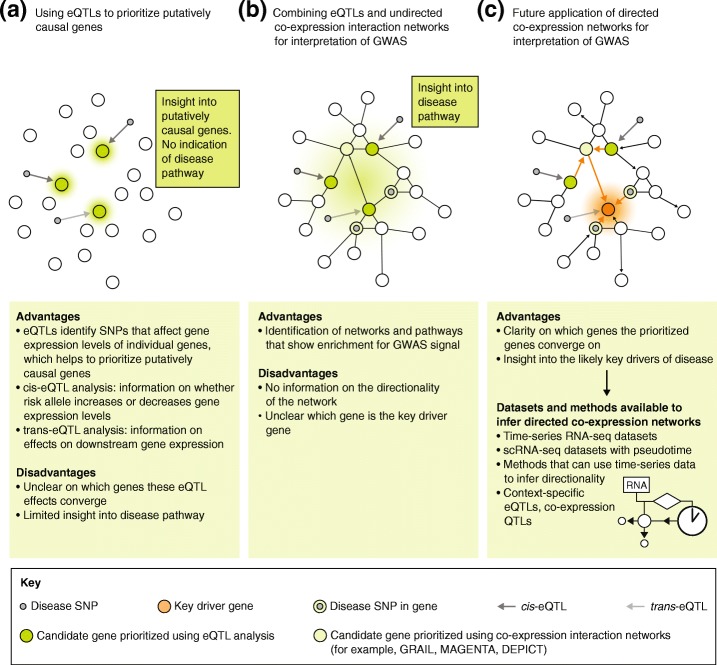
Current and future approaches to understand the role of genetics in disease. **a** To identify putatively causal genes, GWAS SNPs are linked to gene expression using eQTL analysis. **b** To obtain greater understanding of disease pathogenesis, it is essential to look beyond the disruption of individual genes and identify potential disease-associated pathways. This can be done by identifying the co-expression relationships between genes in all loci linked to a specific disease, for example, using methods such as GRAIL [42], MAGENTA [43], and DEPICT [39]. **c** In the future, to pinpoint disease-relevant key driver genes, directional co-expression networks can be generated using a combination of current and novel approaches, including pseudotemporal ordering of scRNA-seq data and context-dependent eQTL and co-expression QTL analysis. *eQTL* expression quantitative trait locus, *GWAS* genome wide association studies, *scRNA* single-cell RNA, *SNP* single nucleotide polymorphism

In the past decade, genome-wide association studies (GWAS; Box 1) have identified over 10,000 genetic risk factors, mainly single nucleotide polymorphisms (SNPs), for more than 100 common diseases [1]. Together these GWAS loci can explain up to 25% of the heritability of complex diseases [2] and up to 56% of disease-related traits [3]. The majority of these genetic risk factors are located in non-coding regions [4] and, as the function of these regions is challenging to decipher, it remains largely unclear how the SNPs are linked to disease. Several studies have shown that the gene nearest to the genetic association may not always be the causal gene [5–7]. Consequently, more sophisticated approaches have been developed to unravel the link between genetic risk factors and disease (for example, by identifying the disease-causing cell types, genes, and pathways; Fig. 1). Expression quantitative trait loci (eQTL) studies, for example, have been performed to identify the local (*cis*-eQTL) and distal (*trans*-eQTL) downstream effects of genetic variation on gene expression [8, 9]. These eQTL studies have provided the first clues about how genetic variation is linked to disease (Fig. 2a). Other methods to further prioritize putatively causal genes include co-localization analysis, fine-mapping, and summary-data-based Mendelian randomization (for detailed discussions of these techniques see [10, 11]). To provide a greater understanding of gene regulatory mechanisms, several large consortia—including the ENCODE project [12], FANTOM [13], Epigenome Roadmap [14], and Blueprint [15]—have systematically classified more than 80% of the genome as non-coding regulatory elements. Genetic variation has now been linked to many of these elements, including epigenetic marks [16, 17], transcription factor binding and chromatin accessibility [18, 19], and post-transcriptional regulation [20, 21].

Studies to date have emphasized the importance of studying both gene expression [22] and its regulation. However, despite these advances in our understanding of GWAS variants, a recent study of 7051 samples from 449 donors across 44 tissues from the Genotype-Tissue Expression (GTEx) project linked only 61.5% of the SNPs within a GWAS locus to an eQTL effect [23]. The reason that not all GWAS SNPs can be linked to an eQTL effect could be that eQTL studies have been performed in the wrong context for a specific disease. We now know that many genetic risk factors have cell-type-specific effects [22, 24, 25] or are modulated by environmental factors [26, 27] and these are contexts that eQTL studies usually do not completely capture.

**Fig. 3:**
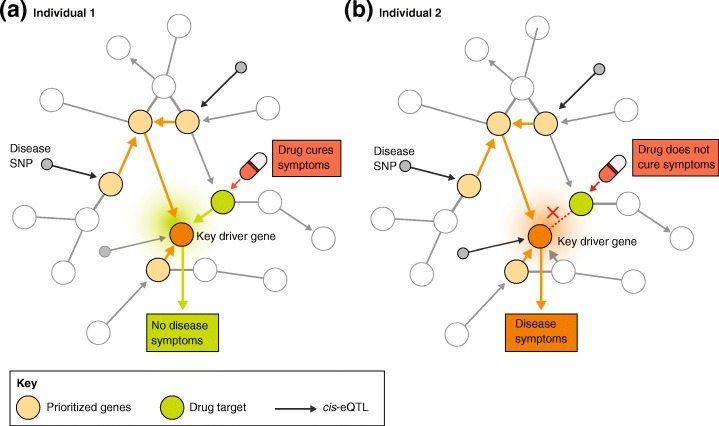
Implications of personalized gene regulatory networks for precision medicine. Depending on an individual’s regulatory wiring, specific drugs may or may not be effective. Personalized GRNs will provide guidance for precision medicine in the future. In this example, GRNs of two hypothetical patients are shown in which the regulatory wiring between the drug target gene and the key driver gene is different. **a** In individual 1, the drug target gene activates the key driver gene. **b** In individual 2, the interaction between both genes is absent. Thus, in individual 1, the drug is effective, whereas in individual 2, the drug is ineffective. *GRN* gene regulatory network

Independent genetic risk factors can converge into key regulatory pathways [24, 28] and may act beyond the disruption of individual genes [29, 30]. Therefore, we expect that a comprehensive overview of the many processes at work will be required to better understand disease pathogenesis. This kind of overview can be acquired by reconstructing gene regulatory networks (GRNs) that are based on cell type [22, 24, 25], environment [26, 27], and an individual’s genetic makeup [29, 30]. A GRN is a directional network of genes in which relationships between genes and their regulators are mapped. Understanding the effect of genetic variation on GRNs is particularly important because this may contribute to the large inter-individual variation in drug responsiveness (Fig. 3). At present, some of the most commonly prescribed drugs are effective in only 4 to 25% of the people for whom they are prescribed [31].

Here, we outline our vision for an integrative approach to reconstruct context-specific GRNs. We focus on gene expression-based regulatory networks because a wealth of gene expression data is already available and the generation of this type of data at the bulk and single-cell levels has advanced the most compared to other single-cell technologies. However, there are other molecular levels, such as metabolites or proteins, which should be included in GRNs in the future to capture the full complexity of a disease [32].

We begin with a brief introduction to the concept of a co-expression network and describe the methods used to create directional GRNs from co-expression networks using bulk data. We then discuss the limitations of bulk data and how these can be resolved by the unique properties of novel single-cell gene expression approaches to enable the reconstruction of causal GRNs. Methods used to reconstruct single-cell GRNs have been reviewed recently by Fiers et al. [33] and are therefore not covered in detail here. We conclude by describing how the combination of bulk and single-cell data can be used to reconstruct context-specific, personalized GRNs, and describe their use in advancing personalized healthcare.

## Gene networks in bulk data

Understanding the pathways affected in disease requires a clear definition of which genes act together in specific cellular processes. To this end, model organisms have been instrumental in defining the most basic pathways present in each cell. By performing knockout experiments, for instance, the relationships between genes can be identified by studying the downstream effects on gene expression or enzymatic function, and these effects are now catalogued in databases such as KEGG [34] and REACTOME [35]. The pathways defined in these databases, however, can be incomplete or biased towards well-studied cellular phenotypes or genes. Co-expression networks and GRNs can therefore be used to extend the knowledge provided by such databases, and methods for constructing such networks have been reviewed in detail elsewhere [36, 37].

Gene networks can be used to infer the functions of unannotated genes by assuming that genes with similar functions are located close together in these networks (i.e. guilt-by-association) [38–42]. The clusters in the network can be overlapped with the genes that are affected by GWAS SNPs to identify the disease-associated pathways, using methods such as GRAIL [42], MAGENTA [43], and DEPICT [39] (Fig. 2b). However, knowing the functions of genes and the associations between genes is often insufficient to identify the key driver gene.

### Implementing directionality in the gene network

Disease-relevant gene clusters can be identified using the methods discussed above, but they do not provide insight into how genetic risk factors affect the network. To identify the downstream consequences of genetic risk factors, directionality must be added to co-expression networks. A GRN is a directional co-expression network that also has information about the regulators that control gene expression. Information obtained from databases such as KEGG [34] can be used to add directionality to specific pathways, but this information is limited in its ability to determine whether these pathways are active in specific cell types or if they function similarly in all cells or individuals. Additional approaches are therefore required to generate context-specific GRNs.

Directionality can be added to a co-expression network using a combination of perturbations, time-series data, and dynamic Bayesian models [44–46] (Fig. 2c; Box 1). However, dynamic Bayesian models cannot be made without time-series data, and generating such data is very costly because it requires a high sampling rate to correctly define directional relationships between genes (which follows from the Nyquist–Shannon sampling theorem that is used to find the sample frequency required to describe a continuous time signal [47, 48]). Undersampling could result in incorrect correlations between genes or in missing key events in the network [49]. Moreover, correct directional inference requires cells to be synchronized to the same cell cycle state before time-series experiments are started, and even when successful, cells may lose their synchronization over time [50]. Finally, the construction of Bayesian networks is computationally intensive [51]. This burden can be reduced by including prior knowledge from other sources (e.g. pathway databases), but the quality of the resulting network will be determined by the reliability of that prior knowledge [52, 53].

**Fig. 4:**
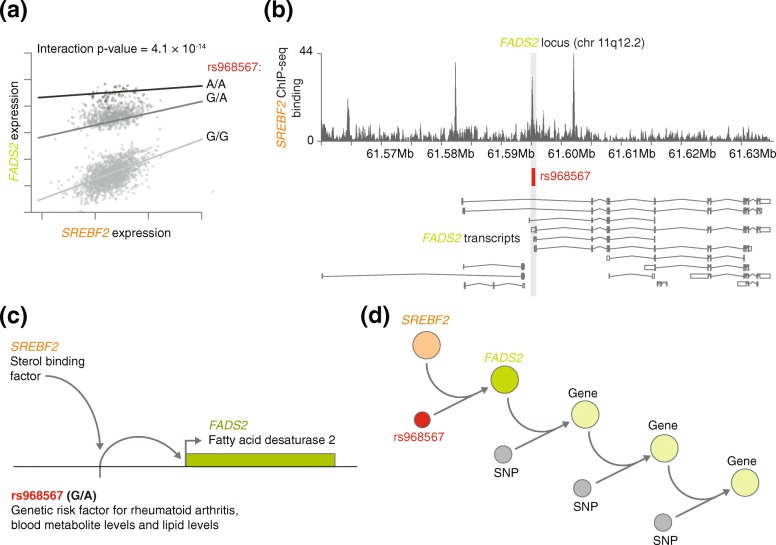
Reconstruction of a gene regulatory network using eQTLs. **a** SNP rs968567 regulates the interaction between the *cis*-regulated eQTL gene *FADS2* and the sterol binding transcription factor *SREBF2* (context-dependent eQTL). **b** ENCODE ChIP-seq data show that this SNP is located within a *SREBF2* binding site, thereby modulating *FADS2* gene expression. **c** Combining the information from *cis*-eQTL and context-dependent eQTL analysis with ChIP-seq information allows us to decipher how SNP rs968567 modulates the expression of the *FADS2* gene. **d** Combining *cis*-, *trans*-, and context-dependent eQTLs or co-expression QTLs has the potential to allow reconstruction of a branch of a gene regulatory network. Parts a–c adapted with permission from Springer Nature, Zhernakova et al. *Nature Genetics* [29], Copyright 2017. *ENCODE* Encyclopedia of DNA Elements, *eQTL* expression quantitative trait locus, *SNP* single nucleotide polymorphism

Information about the regulators that control gene expression can also be gained by linking GWAS variants to additional molecular layers such as transcription factor binding motifs and enhancer and promoter elements [54]. This information can be helpful in determining directionality and understanding how genes are regulated, which provides additional support for putatively causal interactions. Similarly, eQTL information can be linked to additional molecular layers to reveal the functional mechanism of how the genotype affects interactions between genes, so-called context-dependent eQTLs (Fig. 4) [29].

Additional molecular data layers can be very informative for inferring directionality; however, these data are not always available in the disease-relevant context. Recent bulk-based RNA-seq studies have generated tissue-specific co-expression networks for up to 144 different tissues [55, 56]. However, the associated time and cost of implementing directionality and context-specificity have hampered the generation of tissue-specific GRNs in bulk data. In the following sections, we describe how a combination of bulk and single-cell data can be used to resolve these issues and to create GRNs that help us understand the link between genetic risk factors and disease.

## Improving networks with single-cell data

The first single-cell RNA-sequencing (scRNA-seq) experiment was performed with a single cell in 2009 [57]. Since then, the technique has further developed and now more than a hundred thousand cells can be processed in parallel [58, 59]. Recently, efforts have been made to build gene co-expression networks using scRNA-seq data [60–62]. The reliability of these networks improves with increasing numbers of cells, reads-per-gene, and genes-per-cell, but exact numbers are difficult to provide as they are influenced by many factors [61, 62]. We expect that such single-cell-based co-expression networks will be further improved when the consequences of low RNA capture efficiency are overcome [63–65]. One of these consequences is that many PCR cycles are required to generate sufficient material for sequencing, which can result in PCR amplification bias. To overcome this bias, unique molecular identifiers (UMIs; barcodes that tag unique RNA molecules) are added before PCR amplification [66]. However, the most important consequence of low RNA capture efficiency is the high number of dropouts. Dropouts are genes for which no transcript is captured, even though the cell expressed the mRNA. Gene expression imputation has been proposed as a solution for this problem (for a detailed comparison of recent imputation algorithms see [67]). Although several promising solutions have been developed, none have completely solved the problems surrounding the sparseness of single-cell data, and this will likely remain an area of intense study in the coming years.

Most aspects of reconstructing a co-expression network will not differ between single-cell and bulk expression data (reviewed in [33]). However, the assumptions underlying bulk-based network methods on the gene expression distribution (normal distribution) may not apply to single-cell expression data (zero-inflated negative binomial distribution) [68]. The unique features of single-cell data may provide opportunities to enhance the network and will require the development of new algorithms to take these features into account. Ways to enhance the network using single-cell expression data are discussed in the following sections.

### Specifying the context

Gene expression networks change depending on a number of factors, including cell type [22, 24, 25], environment [26, 27], and genetic signature [29, 30], and the influence of each of these contexts can be determined using scRNA-seq.

The ability of scRNA-seq data to dissect complex tissues and detect cell types/states in an unbiased manner [69–71] is valuable for reconstructing cell-type-specific co-expression networks. This kind of dissection using scRNA-seq was recently applied to detect single-cell eQTLs with high resolution [30, 72], which revealed that gene regulation can differ even between cell subtypes [30]. Unbiased classification has also led to the identification of specific cell states and combinations of transcription factors that drive cell-type-specific gene regulatory programs [73]. This study [73] showed that networks are different between brain cell types and that cell-type classification using networks gives better separation than classification based on gene expression levels alone.

Cellular heterogeneity induced by environmental perturbations can also be dissected using single-cell analysis [74]. In the context of co-expression networks, Martins et al. [75] used single-cell qRT-PCR to identify the heterogeneous effects of cytokine stimulations on the rewiring of the network in macrophages. Importantly, some of the effects on the co-expression network they identified would have been overlooked if they had pooled the expression of ten cells; a demonstration of how population-level co-expression networks cannot fully capture gene regulation at the single-cell level.

scRNA-seq can also be used to identify differences induced by genetic variation between individuals, which enables the reconstruction of a person-specific or personalized co-expression network. In contrast to approaches using bulk RNA-seq, it is feasible to generate many measurements per individual with scRNA-seq, which enables the calculation of correlations between genes per individual [30]. These correlations can be used to identify the relationships between genes within a personal co-expression network. This approach was applied recently to identify relationships between genetic variants and the modulation of co-expression in CD4^+^ T cells. Within a cohort of 45 individuals, genetically modulated co-expression relationships, so-called co-expression QTLs, were identified that could be replicated in a bulk RNA-seq dataset of 2116 individuals. However, these relationships would not have been detected using a genome-wide approach in bulk data only [30]. Another advantage of scRNA-seq data is that true correlations between genes can be identified that would otherwise be masked by the effects of averaging in bulk RNA-seq data due to Simpson’s paradox [76] (Box 1).

However, a disease-specific network is not defined by any of the above-mentioned factors (cell type, environment, or genetic signature) alone, but rather by a combination of them. Celiac disease, as an example, occurs only in individuals who carry specific HLA genotypes (genetics) and consume foods containing gluten (environment) [77]. Celiac disease is a well-known example of what is called a ‘genotype by environment (GxE) interaction’, where an environmental perturbation is modulated by an individual’s genetic background. Future scRNA-seq studies should expand our understanding of how genotype by environment interactions modulate co-expression networks, for example, by exposing cells from different individuals to various environmental conditions.

### Exploiting scRNA-seq data variability to infer directionality

**Fig. 5:**
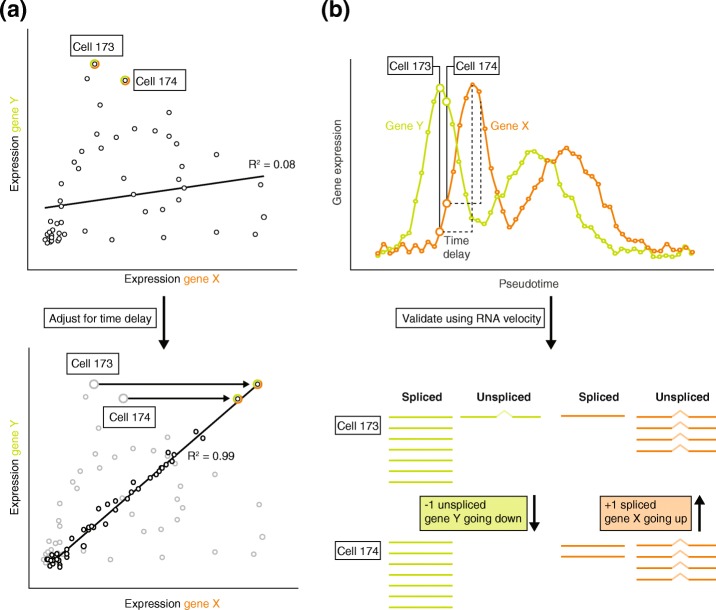
Inferring causality using pseudotime analysis and RNA velocity. **a** In this hypothetical example, when determining the relationship between gene X and gene Y, no correlation would be observed (*top*). However, the relationship between both genes may be masked by a time delay and correcting for this time delay might reveal a clear correlation between the expression of gene X and gene Y (*bottom*). **b** To identify the length of a time delay, the cells can be ordered along pseudotime, that is, an inferred timeline based on the variable gene expression states of single cells measured at a single moment in time (*top*). RNA velocity [86], a readout that exploits the unidirectional character of splicing, allows the prediction of the future state of a cell and its genes (*bottom*). As such, the correct ordering of cells can be validated using RNA velocity. Plotting gene expression against pseudotime shows that the expression of gene X is following the expression of gene Y. From this, it can be deduced that gene Y is regulating gene X, and not the other way around

Measured gene expression levels can vary considerably between different cells even after accounting for cell type, environment, and genotype. The intercellular biological variability in gene expression levels provides unique insights that cannot easily be extracted from bulk expression data. During dynamic processes, such as cell differentiation or a response to an environmental stimulus, cells will move towards another state over time. However, the pace at which cells move into this new state differs between cells. By exploiting the asynchronous nature of these processes between cells, cells can be computationally ordered in pseudotime based on expression similarity [78, 79]. This pseudotemporal ordering of cells can provide temporal resolution in an experiment that does not explicitly capture cells along a time-series. Insights can therefore be gained using scRNA-seq data that would remain hidden in bulk data, while requiring only one snapshot from a single sample (Fig. 5). At present, there are more than 50 different methods for pseudotemporal ordering of cells (see [80] for a recent comparison of these methods).

Pseudotime analyses have been used to reconstruct co-expression networks [81, 82] or small directional GRNs [83] from single-cell data (see [33] for an overview of current computational methods). However, the assumptions required for pseudotemporal ordering of cells are often violated in practice, which can result in incorrect assignment of directionality [84, 85]. The sampling frequency inferred by these methods, for instance, depends on sample size, which could be insufficient to recreate the complete underlying process of interest. Furthermore, several different networks may give plausible explanations for the same observed distribution of cell states. Therefore, it is difficult to determine the correct underlying mechanism of gene regulation without prior knowledge.

Both these issues can be resolved using a method called RNA velocity [86], which exploits the unidirectional character of splicing. RNA velocity examines the rate of change of mRNA molecule abundances in the cell by modeling the relationship between unspliced mRNA (an indicator of current transcription) and spliced mRNA (an indicator of transcription in the recent past). Although 3′-end scRNA-seq data do not cover the entire mRNA transcript, these data as well as full-length data can be used to study RNA velocity. By taking the RNA velocity information of all genes together, a cell’s future state can be successfully predicted [86, 87]. Moreover, RNA velocity artificially enriches the inferred sampling frequency and prioritizes the pseudotemporal order that explains the observed distribution of cell states.

Interestingly, in the context of GRNs, combining the information extracted from RNA abundance and RNA velocity improves the ability to predict true targets of transcription factors across a range of species and for experimental settings that mimic the sparseness and noisiness of scRNA-seq data [88]. Moreover, a time-delay between gene–gene interactions can be implemented to reflect the delay in gene expression changes upon a gene–gene interaction. This was shown to result in greater accuracy to identify time-delayed interactions and infer network topology [89, 90]. As such, similar to application of time-series bulk data, we reason that causality can be inferred in GRNs using a combination of RNA velocity and pseudotemporal ordering (Fig. 5).

## Integrative approach for GRN assembly

**Fig. 6:**
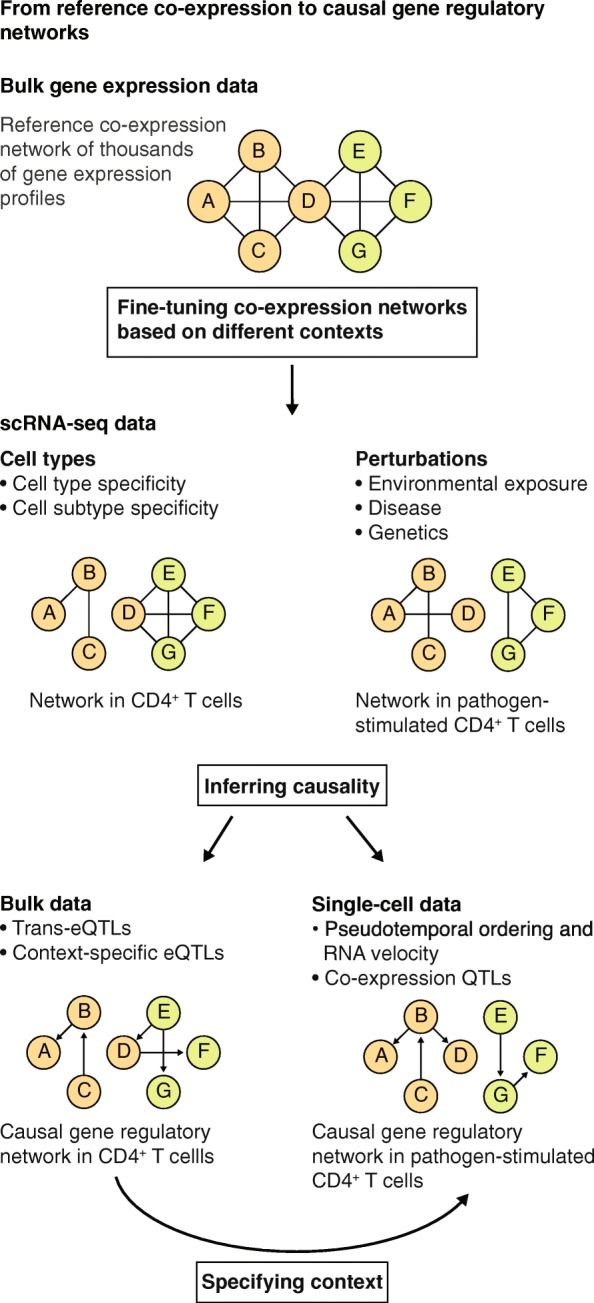
Reconstruction of personalized, context-specific gene regulatory networks through the integration of bulk and single-cell data. We expect the richness of bulk expression data (for example, the number of genes or transcript variants detected and the number of datasets available for any given tissue) combined with the context-specificity of scRNA-seq data (for example, cell type and environmental exposure) will facilitate the generation of context-specific co-expression networks. Finally, integrating additional data layers, such as context-specific eQTLs and co-expression QTLs combined with ChIP-seq data, will allow the direction of effects to be determined. This information will enable the reconstruction of personalized, context-specific gene regulatory networks for use in precision medicine

Considering the unique features and applicability of both bulk and scRNA-seq data for generating GRNs, we propose using an integrative approach to assemble context-specific, personalized GRNs that can help move towards improved precision medicine in the future. This integrative approach combines the richness of bulk data with the finer detail and unique insights obtained from single cells (Fig. 6). Our proposed approach consists of an interplay alternating between bulk and single-cell data, iteratively updating GRNs with knowledge acquired from both sources of data. This allows us to take full advantage of both technologies and recent collaborative efforts, such as the Human Cell Atlas [91], the GTEx consortium [22], and the single-cell eQTLGen consortium [92]. In the following sections, we describe the three steps of this integrative approach using the example of hypothetical CD4^+^ T-cell data illustrated in Fig. 6.

### Bulk-based reference co-expression network

The first step in assembling a context-specific GRN is establishing a cell-type-specific reference network that can be used as a baseline onto which the specific contexts can be projected. To create this reference network, numerous publicly available datasets for specific cell types made with bulk RNA-seq can be used. Public RNA-seq repositories, such as the European Nucleotide Archive [93] and the Sequence Read Archive [94], already contain hundreds of bulk RNA-seq datasets from purified cell types. Combining these datasets from different resources requires uniform alignment, quantification, and removal of batch effects [95], and several recent efforts have combined such uniformly processed bulk RNA-seq datasets in large repositories [96–99]. Based on previous benchmarking studies (comparing the performance of network reconstruction approaches against a known reference network) using both bulk and in silico data, community-based approaches seem most suitable for reconstructing such reference networks [100].

Although single-cell data provide a more detailed context of the network, at present they do not have the sensitivity of bulk data and will create an incomplete network due to dropouts. The bulk reference co-expression network thus serves two purposes. The first is to fill gaps in the network where expression, and therefore any possibility of an interaction, is missing for genes. The second is to provide additional supporting information when evidence from single-cell expression data is insufficient to confidently report the interaction between two genes. In this scenario, comparison between the single-cell and bulk RNA-seq reference can be used to gain additional support for the interaction.

To successfully use a bulk-based reference network, stable parts of the network, so-called anchor points, have to be identified. Anchor points are parts of the network that are shared between the reference network and the personalized single-cell network. With the bulk-based reference network as a basis on which the single-cell data can be projected, further context-specific connections can be investigated.

### Fine-tuning the reference co-expression network to reflect the context

The second step in assembling the context-specific GRN is to use scRNA-seq data to add context-specific information to the bulk-based reference co-expression network. Single-cell data enable sampling to be performed on a whole tissue, after which individual cell types can be dissected using the single-cell expression profiles. This allows for the creation of cell-type-specific networks without the need to predefine the studied cell types. Furthermore, for each of the identified cell types, the effect of environmental perturbations can be studied. To illustrate this second step, we provide a hypothetical example in Fig. 6 of a CD4^+^ T-cell-specific and pathogen stimulation-perturbed network. By generating such a network for each individual separately, the higher complexity of the network can be captured.

Several single-cell-specific computational models have been developed to generate GRNs that could be used for this purpose [33]. Such models are required to correct for dropouts and to take the single-cell-specific gene expression distribution into account [68, 101]. Nevertheless, benchmarking revealed that both general bulk-based and specific single-cell-based approaches showed poor performance using experimental and in silico single-cell data [68]*.* Benchmarking of these algorithms remains a challenge due to the lack of a gold standard network for comparison. The comparison network used at present is based on bulk data, and consists of interactions found in a combination of many cell types and contexts together resulting in a notable difference between bulk gold standard networks and networks derived from single-cell data [68]. This may be because interactions found in bulk-based reference networks are not truly representative of interactions found at the single-cell level.

An experimentally validated single-cell gold standard network will advance the development of single-cell-specific network reconstruction algorithms. We believe that pooled CRISPR-screens coupled with a scRNA-seq readout, such as CROP-seq [102], CRISP-seq [103], and PERTURB-seq [104, 105], offer the possibility to create such a single-cell-based gold standard network. These methods enable mapping of the downstream consequences of gene perturbations on the whole transcriptome level. Not only can these experimental methods be used to create a gold standard, they can also provide insights into causal gene–gene relationships.

### Transitioning from associations to causal relationships

The final step in assembling the GRN is to add directionality to the context-specific network to gain insight into the putatively causal relationships between genes and to validate them using experimental approaches. Our proposed method utilizes easily accessible data to solve the issue of directionality by integrating information from eQTLs or pseudotemporal ordering into the network.

There are several ways in which eQTLs can be used to gain insight into the GRN. First, they can reveal downstream effects of gene regulation. SNPs that have both *cis* and *trans* effects on gene expression can be used to uncover regulatory relationships between genes. For example, Westra et al. [24] have shown that such SNPs may affect the expression of a transcription factor in *cis* and consequently affect the expression of many downstream genes in *trans*. For a number of these downstream genes supporting ChIP-seq data were found, which suggest directionality of regulation. Second, context-dependent eQTLs [29] and co-expression QTLs [30] can uncover the upstream interactors of some genes and identify parts of the network where the relationships between genes change with genotype (Fig. 4). Altogether, by combining *cis*-, *trans*-, and context-dependent eQTLs or co-expression QTLs, branches of a GRN can be reconstructed and extended with genetic information.

To put the regulatory information obtained from eQTLs into the correct context, the cell types in which the eQTL effects manifest have to be identified [22, 24, 25]. Identification of *trans*-eQTLs and context-dependent eQTLs requires enormous sample sizes in bulk expression data (thousands of samples) to overcome a severe multiple-testing burden [24, 29]. Such massive datasets are currently only available for whole tissues in bulk (such as whole blood), but these do not allow identification of the relevant cell type. Although the sample size of single-cell datasets does not permit these analyses at the genome-wide level, single-cell datasets can be used to determine the cell type in which an eQTL effect identified from bulk data manifests. After pinpointing the relevant cell type, bulk multi-omics data of this specific cell type can be used to identify or verify the regulating mechanism behind the context-dependent interaction. For example, a genetic variant was shown to change enhancer–promoter looping by affecting the affinity of a cell-type-specific transcription factor [106]. By providing this kind of evidence for the regulating mechanism, causality can be integrated into the parts of the cell-type-specific GRN for which eQTLs can be found.

Combining pseudotemporal ordering with RNA velocity allows the identification of directionality between all genes, not just eQTL genes. Identifying which genes share similar expression patterns and the pseudotime at which they are expressed can establish the directional relationship between these genes (Fig. 5). van Dijk et al. [107] recently showed promising results with a comparable approach in which imputed gene expression scRNA-seq data were ordered along pseudotime. Subsequently, transcription factors and potential targets that change considerably along pseudotime were selected. In this way, they reconstructed a large GRN consisting of 719 transcriptional regulators and 11,126 downstream target genes. Of the predicted target genes that were tested, 92% significantly overlapped with target genes as assessed by ATAC-seq (assay for transposase-accessible chromatin using sequencing). This study showed promising results to identify target genes without experimental perturbation. However, experimental validation is required to transition from associations to causal relationships. Our proposed integrative approach will help to provide focus on those regions of the network that are of particular interest and alleviates the need to perform experimental validation on every possible gene, thereby circumventing the high cost associated with testing all combinations. Altogether, we expect that such an integrative approach will enable the reconstruction of well-validated context-specific, personalized GRNs.

## The future of precision medicine

A major challenge in healthcare today is that the majority of prescribed drugs are only effective in a small subset of patients [31]. This not only leads to money lost on ineffective drugs, but it also unnecessarily exposes patients to adverse drug side effects. Well-validated, context-specific, personalized GRNs will be essential to move from more traditional medicine towards precision medicine, which will provide treatment or preventive measures that will be effective for patients based on their specific genetic, environmental, and lifestyle characteristics (Fig. 3). In order to successfully implement the proposed ideas, several technical and practical challenges have to be overcome (Table 1). Overcoming these challenges will open the road for implementing GRNs for precision medicine.

**Table 1:** Challenges associated with implementation of the proposed integrative approach for precision medicine

	Challenge	Solution	References
Technical challenges	Implementation of directionality and causality	eQTL, context-dependent eQTL and co-expression QTL information Time-series data and pseudotime combined with RNA velocity Experimental validation using CRISPR perturbations coupled to scRNA-seq read-out (for example, CRISP-seq, CROP-seq, and PERTURB-seq)	[24, 29, 30, 86, 102–105]
	Dropouts	Gene expression and cross-omics imputation	[67, 118, 119]
	Amplification bias	Unique molecular identifiers (UMIs)	[66]
	Combining single-cell data with a bulk reference network	Anchor points Computational methods need to be developed	[120]
Practical challenges	Time and cost involved in collecting scRNA-seq data	Droplet-based approaches in combination with approaches that enable super-loading and pooling of samples (for example, cell hashing or demuxlet) Split-pool barcoding approaches (for example, SPLiT-seq and combinatorial indexing) Large throughput sequencers that enable reduction in sequencing cost	[58, 59, 72, 121–124]
	Large-scale availability of datasets with both genotype and scRNA-seq data	Collaborative efforts (for example, single-cell eQTLGen consortium and Human Cell Atlas)	[91, 92]
	Cost involved in genotyping each individual	Genotype arrays in combination with imputation-based approaches enable mapping of clinically relevant genetic variants with high coverage for less than €100 per individual	[117, 125, 126]
	Public perception, health regulations	General Data Protection Regulation implemented in the EU in 2018 Genetic counselors to help with interpreting genetic results	[113]

Disease-specific GRNs may provide novel insights into disease pathogenesis and have enhanced power to prioritize disease-causing genes [108]. These GRNs provide a bird’s-eye view to look beyond the disruption of individual disease genes: each gene may have a small individual effect, but several disease genes together may have a large additive effect when converging into a few disrupted key regulatory pathways [109–111]. Despite the involvement of different individual disease genes, similar key regulatory pathways are likely to be disturbed in several different diseases. Likewise, exposure to specific environmental factors may disturb regulatory pathways in a fashion comparable to specific disease-associated genetic variants. These insights may provide novel links between different diseases or clues to how environmental factors can contribute to one or more diseases, and these new associations should provide novel directions for treatment.

Generation of context-specific GRNs may never fully capture the complexity of multifactorial interactions (for example, genetic background, environmental exposures, and disease) and the intercellular communication that influences the whole organism. Nevertheless, GRNs will be valuable for predicting the outcome of perturbations, and this particular function of GRNs will be useful for predicting potential drug targets for disease. Tumor-specific networks inferred using a combination of gene expression data and cancer-related signaling pathways have already been successfully applied to identify oncogenes and previously identified targets of cancer treatment [112].

An integral component for disease treatment based on a personalized GRN is to have a patient’s genotype information available. Genotyping patients may allow doctors to select effective drugs while preventing unnecessary adverse effects for the patient. However, before this can be implemented in clinical practice, a shift in both public perception and healthcare regulations is required. For example, updated privacy and data protection regulations, such as the General Data Protection Regulation implemented in the EU in 2018 [113], will be important to reduce privacy concerns in the general public, as this puts individuals in control of their own data. With these recent developments in policy and public perception it is becoming more likely that more people will be genotyped, which will help to build personalized GRNs that can be used in precision medicine.

## Conclusions and future perspectives

We have highlighted the importance of using a gene network-based approach rather than a single-gene focused approach to gain the bird’s eye view required to understand disease pathogenesis. As diseases arise in highly specific contexts, context-dependent GRNs are needed to fully understand these diseases. To build these context-dependent GRNs, we have proposed an integrative approach of generating GRNs using both bulk and single-cell data. We have not described the computational implementation of our approach, as this would go beyond the scope of this article. Nevertheless, we expect that our iterative approach is well-suited to implementation using machine learning or deep learning models that learn from large datasets and make predictions on likely outcomes of complex cellular systems such as GRNs [114, 115]. This requires generating massive datasets for which the first steps are being taken in consortia such as single-cell eQTLGen [92] and the Human Cell Atlas [91]. These datasets will be instrumental for executing our integrated approach using machine learning algorithms. Moreover, platforms such as the Human Cell Atlas are expected to provide more uniform guidelines and solutions for generating, processing, and handling large-scale scRNA-seq data. This will facilitate the combining of scRNA-seq datasets as part of our integrative approach.

As initiatives such as 23andMe [116] and the UK Biobank [117] produce ever larger genetic datasets that could be used to reconstruct personalized GRNs, and new initiatives are started, the ability to accurately predict disease risk through a combination of genotype associations and personalized GRNs will improve. However, before these personalized GRNs can be adopted in clinical practice, a number of ethical and legal issues will have to be resolved. Clinical guidelines, for instance, will have to be developed so that the interpretation of the results can be guided by trained professionals and the actionability of individual findings has to become clear [32]. Once these issues have been addressed, we expect that personalized, context-dependent GRNs will accelerate the progress required to make the next big leap in precision medicine.

## Box 1. Glossary

Bayesian model: statistical modeling to calculate probabilities for an observation while taking into account the empirical or theoretical expected distribution of these observations or factors expected to influence the observations. Used in co-expression networks to assign probabilities for directionality between genes.

Benchmarking: comparing the performance of a computational model against a gold standard or known solution.

Co-expression network: an undirected network that describes which genes often behave in a coordinated manner. The network consists of nodes, representing genes, that are connected through edges that represent relationships between nodes. These relationships can be unweighted or weighted, indicating either a binary relationship (on/off) or a more continuous relationship.

Co-expression QTLs: SNPs that modulate the correlation between the co-expression of two genes. To identify these, many observations (for example, on multiple cells or tissues) per individual are required.

Co-localization: a method that determines whether the association signals in a locus correspond between two association studies (for example, between a GWAS and an eQTL study).

Context-dependent eQTLs: eQTLs for which the strength of association depends on a secondary factor. This may be either intrinsic (for example, expression of another gene or cell type frequency) or extrinsic (for example, environmental exposure). Gene expression data can be used as proxy measurements for both intrinsic and extrinsic factors.

Dropouts: genes that fail to be detected even though they are expressed (resulting in a zero-inflated gene expression distribution) due to incomplete mRNA capture by current scRNA-seq technologies.

Expression quantitative trait loci (eQTL): a SNP that explains a variation in gene expression levels. When the SNP explains the expression of a gene within a 1-megabase distance, it is called a *cis*-eQTL. When the SNP explains the expression of a gene beyond a 1-megabase distance, it is called a *trans*-eQTL.

Fine-mapping: a statistical approach that is used to prioritize the most likely causal genetic variant in a previously identified locus that is linked to a specific phenotype.

Gene regulatory network (GRN): a directional co-expression network that also contains information about the regulators that control gene expression.

Genome-wide association studies (GWAS): genome-wide approach in which genetic variants such as single nucleotide polymorphisms (SNPs) are linked to a molecular trait or disease.

Genotype by environment (GxE) interactions: interactions between an individual’s genotype and the environment. Context-dependent eQTLs are a subset of GxE interactions.

Machine learning approaches: methods used to analyze massive amounts of data in order to build predictive models from multi-dimensional datasets.

Nyquist–Shannon sampling theorem: describes the sample frequency that is sufficient to capture all the information from a continuous-time signal of a finite bandwidth.

Precision medicine: healthcare that is individually tailored on the basis of a person’s genetic, environmental, and lifestyle characteristics.

Pseudotime: temporal sequences of gene expression states in cells inferred from measurements made at a single moment in time.

RNA velocity: the rate of change of mRNA molecule abundances in the cell determined by modeling the relationship between unspliced mRNA (an indicator of current transcription) and spliced mRNA (an indicator of transcription in the recent past).

Simpson’s paradox: a situation in which an observed relationship within different samples (or groups of data) disappears or reverses when the samples (or groups) are combined.

Summary-data-based Mendelian randomization (SMR): a summary statistics based variant of Mendelian randomization that leverages the principle that genetic variation is randomly assigned to a sample with a specific phenotype to infer causality between genetic variation and the phenotype in an observational study.

Unique molecular identifiers (UMIs): barcode sequences tagging individual molecules.

## Abbreviations

eQTL: Expression quantitative trait locus
GRN: Gene regulatory network
GTEx: Genotype-Tissue Expression project
GWAS: Genome-wide association study
GxE: Genotype by environment
scRNA-seq: Single-cell RNA-sequencing
SNP: Single nucleotide polymorphism
UMI: Unique molecular identifier
